# Baicalin Attenuates Hypoxia-Induced Pulmonary Arterial Hypertension to Improve Hypoxic Cor Pulmonale by Reducing the Activity of the p38 MAPK Signaling Pathway and MMP-9

**DOI:** 10.1155/2016/2546402

**Published:** 2016-09-01

**Authors:** Shuangquan Yan, Yiran Wang, Panpan Liu, Ali Chen, Mayun Chen, Dan Yao, Xiaomei Xu, Liangxing Wang, Xiaoying Huang

**Affiliations:** ^1^Division of Respiratory Medicine, Taizhou Enze Medical Center Enze Hospital, Taizhou, Zhejiang 318000, China; ^2^Division of Equipment, The First Affiliated Hospital of Wenzhou Medical University, Wenzhou, Zhejiang 325035, China; ^3^Division of Intensive Care Unit, Ningbo Medical Center Lihuili Eastern Hospital, Ningbo, Zhejiang 315040, China; ^4^Division of Pulmonary Medicine, The First Affiliated Hospital of Wenzhou Medical University and Key Laboratory of Heart and Lung, Wenzhou, Zhejiang 325035, China

## Abstract

Baicalin has a protective effect on hypoxia-induced pulmonary hypertension in rats, but the mechanism of this effect remains unclear. Thus, investigating the potential mechanism of this effect was the aim of the present study. Model rats that display hypoxic pulmonary hypertension and cor pulmonale under control conditions were successfully generated. We measured a series of indicators to observe the levels of pulmonary arterial hypertension, pulmonary arteriole remodeling, and right ventricular remodeling. We assessed the activation of p38 mitogen-activated protein kinase (MAPK) in the pulmonary arteriole walls and pulmonary tissue homogenates using immunohistochemistry and western blot analyses, respectively. The matrix metalloproteinase- (MMP-) 9 protein and mRNA levels in the pulmonary arteriole walls were measured using immunohistochemistry and in situ hybridization. Our results demonstrated that baicalin not only reduced p38 MAPK activation in both the pulmonary arteriole walls and tissue homogenates but also downregulated the protein and mRNA expression levels of MMP-9 in the pulmonary arteriole walls. This downregulation was accompanied by the attenuation of pulmonary hypertension, arteriole remodeling, and right ventricular remodeling. These results suggest that baicalin may attenuate pulmonary hypertension and cor pulmonale, which are induced by chronic hypoxia, by downregulating the p38 MAPK/MMP-9 pathway.

## 1. Introduction

Pulmonary arterial hypertension (PAH) is characterized by pulmonary vasoconstriction and lung circulation remodeling, which can gradually elevate pulmonary vascular resistance, leading to right ventricular hypertrophy, dilatation, and dysfunction. Chronic hypoxic exposure can induce PAH, eventually leading to right ventricular hypertrophy and failure. Pulmonary arteriole remodeling, which includes smooth muscle cell proliferation, extracellular matrix (ECM) turnover, and collagen fiber accumulation, is the key step in this process [1]. Matrix metalloproteinase- (MMP-) 9 can participate in ECM turnover, fibrosis, and chronic inflammation, and MMP-9 promotes the proliferation of smooth muscle cells in blood vessels and their migration into the vessel wall [2, 3]. The same process occurs in the small pulmonary artery.

As a member of the mitogen-activated protein kinase (MAPK) family, p38 MAPK can be activated by the phosphorylation of its subunits, and this activation plays an important role in inflammation and cell differentiation and proliferation in arteries [4, 5].

Enhanced p38 MAPK activation can upregulate the level of MMP-9 by promoting MMP-9 mRNA transcription levels, which then leads to a series of biological effects [6].

Baicalin is a flavonoid compound purified from the dry roots of* Scutellaria baicalensis*, and it possesses a variety of pharmacological activities, such as antioxidative, anti-inflammatory, antitumor, and antiapoptotic activities; it also inhibits smooth muscle cell proliferation [7–9]. We previously found that baicalin attenuates small pulmonary artery remodeling in vivo in rats with hypertension [10], but the mechanism is complicated and not fully understood. Several studies have shown that baicalin downregulates the expression of p38 MAPK and MMP-9. Baicalin not only inhibits the expression of p38 MAPK and MMP-9 in new retinal vessels in a mouse model of oxygen-induced retinopathy [11] but also blocks the activation of p38 MAPK, which leads to reduced MMP-9 levels and tumorigenicity in MDA-MB-231 cells [12]. Thus, we speculate that baicalin attenuates pulmonary arteriole remodeling and hypoxic pulmonary hypertension, further improving cor pulmonale by inhibiting the activity of p38 MAPK and downregulating the expression of MMP-9 in arterioles.

## 2. Materials and Methods

### 2.1. Animals

Twenty-four male specific-pathogen-free Sprague-Dawley rats weighing 220–250 g provided by the Laboratory Animal Center of Wenzhou Medical University were used in the present study. All rats were housed in controlled conditions and fed a standard diet. The animal experiments were approved by the Laboratory Animal Ethics Committee of Wenzhou Medical College and performed in strict accordance with the guidelines for animal ethics.

### 2.2. Drugs and Antibodies

Baicalin (CAS number: 21967-41-9, Product number: 572667, purity 95%) was purchased from Sigma-Aldrich Co., Ltd. (USA). A 2% baicalin solution was prepared by mixing 1 g of baicalin into 48 mL of sterile saline and titrating the solution under stirring with sodium hydroxide until reaching a pH of 6.8–7.0. After the baicalin was completely dissolved, the clear, yellow-green solution was brought up to a final volume of 50 mL with sterile saline, and the final concentration was 20 mg/mL. Then, aliquots of the solution were prepared and stored at −20°C. Prior to use, these aliquots were gradually thawed at 4°C. The rabbit anti-rat p-p38 MAPK polyclonal antibody (#4631) was obtained from Cell Signaling Technology (USA). The secondary horseradish peroxidase- (HRP-) conjugated goat anti-rabbit antibody and the secondary rabbit anti-goat antibody were purchased from ZSGB Biotechnology Co., Ltd. (Beijing, China). The goat anti-rat MMP-9 antibody (sc-6840) was obtained from Santa Cruz (USA). The MMP-9 mRNA in situ hybridization (ISH) kit (MK1540) was purchased from Boster Biotechnology Co., Ltd. (Wuhan, China), and the rabbit anti-rat GAPDH antibody was obtained from Goodhere (China).

### 2.3. Animal Grouping and Administration

The rats were randomized into the following three groups, with eight rats per group: the control group, the hypoxia group, and the baicalin + hypoxia group. The rats in the hypoxia group were kept in a normobaric hypoxic chamber for 4 weeks with an O_2_ concentration of 8–10% and a CO_2_ concentration that was below 5% for 6 days per week and 8 hours per day. The rats in the baicalin + hypoxia group were intraperitoneally injected with 2% baicalin (30 mg/kg) 30 min before being placed into the normobaric hypoxic chamber. The rats in the control group were kept under normobaric and normoxic conditions.

### 2.4. Mean Pulmonary Artery Pressure (mPAP) and Mean Systemic Artery Pressure (mSAP) Measurements

After 4 weeks, the rats were weighed and anesthetized by an intraperitoneal injection of 5% chloral hydrate (40 mg/kg of body weight). The specific methods used have been previously reported by our team. A small skin incision was made in the neck of each mouse. Then, the right external jugular vein was isolated, and a polyethylene catheter (inner diameter, 0.9 mm; outer diameter, 1.1 mm) with a slightly curved front was gradually inserted into the right atrium and then the right ventricle (RV). After the recorder entered the RV, the large vibrations of the ventricular waveform could be documented. Then, the tube was further inserted by approximately 0.5 cm. After the heart rate became normal and the waveform stabilized, the tube was rotated clockwise to the lower part of the mark such that the tube head was facing upward, thereby enabling it to move forward 1-2 cm and enter the pulmonary artery. At this point, the effects of breathing on the pulmonary artery waveform were visible. After moving the tube forward an additional 0.5–1 cm, stable pulmonary artery waveforms could be recorded, and the mPAP was recorded with a piezoelectric pressure transducer and the PowerLab system (AD Instruments, Australia).

### 2.5. Pulmonary Arteriole Remodeling Measurement

Lung tissue sections were subjected to hematoxylin and eosin (H&E) staining following standard histological procedures. For each section, five vessels near the pulmonary alveoli with diameters of approximately 50–200 *μ*m were randomly selected for measurement. First, the pulmonary arteriole wall area and total area (microscope magnification: 40 × 10 = 400x) were measured to calculate the ratio of vessel wall area to total area (WA/TA). Second, each arteriole wall thickness (WT) and radius (*R*) were measured at 0°, 90°, 180°, and 270° by drawing a line across the vessel and its wall. The WT and* R* values acquired from the four angles were then averaged and used to calculate the WT/*R* ratio. Finally, the number of nuclei in the arteriole wall was counted and used to calculate both the ratio of the number of nuclei to the vessel WA and the nuclear density of the wall. All measurements were performed using Image-Pro Plus software (Media Cybernetics, Bethesda, MD, USA).

### 2.6. Right Ventricular Hypertrophy Measurements

The left ventricle plus the interventricular septum (LV + S) and the RV were first collected by cutting along the edge of the RV and the interventricular septum; then, these samples were weighed. The mass ratios of the RV to the LV + S and rat body weight (BW), expressed as RV/(LV + S) and RV/BW, respectively, were used to reflect the degree of right ventricular hypertrophy. Each RV was then immediately placed in 4% formalin, where it was kept for 48 hours before being embedded in a paraffin block. Subsequently, 3 *μ*m thick sections were prepared and stained with H&E to identify the myocardial tissue.

### 2.7. Immunohistochemical Analysis of p38 MAPK Phosphorylation

The sections were deparaffinized, dehydrated, immunostained with the primary rabbit anti-rat p-p38 MAPK polyclonal antibody (1 : 25), and then incubated in the secondary HRP-conjugated goat anti-rabbit antibody (1 : 10000). Negative controls were incubated in phosphate-buffered saline (PBS) in place of the specific primary antibody. All antibodies used for immunohistochemistry were diluted in PBS. Immunoreactivity was visualized using 3,3′-diaminobenzidine (DAB), and immunohistological images were obtained using an intelligent biological microscope (Leica DM1000, Germany). Five fields of view were randomly selected from each section for quantitative analyses. The integrated optical density (IOD) of the positive products in the pulmonary arteriole and the arteriole area were both measured using Image-Pro Plus software; then, the ratio of the IOD to the arteriole area was calculated to reflect the expression of positive products.

### 2.8. Immunohistochemical Analysis of MMP-9 Expression

After being blocked, the sections were incubated with the primary goat anti-rat MMP-9 antibody (1 : 50) and then with the secondary rabbit anti-goat antibody (1 : 10000). Negative controls were prepared by incubating sections in PBS instead of the specific primary antibody. Immunoreactivity was visualized using DAB. All antibodies used for immunohistochemistry were diluted in PBS, and five fields of view were randomly selected from each section for quantitative analyses by Image-Pro Plus software.

### 2.9. ISH for MMP-9 mRNA Measurements

Paraffin-embedded sections were deparaffinized and dehydrated through a graded ethanol series, and they were then processed using a commercially available MMP-9 mRNA ISH kit (MK1540) in accordance with the manufacturer's instructions. The sections were then subjected to a chromogenic reagent. The reaction was visually monitored and stopped by placing the slides in water. The slides were counterstained with a red fixed-nucleus reagent to visualize the nuclei; then, the slides were washed with water, dehydrated, vitrified with dimethylbenzene, mounted in neutral balata, and covered with coverslips. Negative controls were prepared by being hybridized without probes in parallel with the experimental reactions. Images were captured and saved using an intelligent biological microscope, and five fields of view were randomly selected from each section for quantitative analysis by Image-Pro Plus software.

### 2.10. Western Blot Analysis of p38 MAPK Phosphorylation

The protein concentration of the fresh lung tissue was measured using the Bradford method; equal amounts of protein were separated by electrophoresis and transferred onto a polyvinylidene fluoride membrane (Millipore, MA). Nonspecific antibody binding was blocked by incubating the membrane with 5% bovine serum albumin in Tris-buffered saline with Tween-20 (TBST) for 1 h. Subsequently, the membrane was incubated overnight at 4°C with specific rabbit anti-rat p-p38 MAPK polyclonal (1 : 1000) and rabbit anti-rat GAPDH antibodies (1 : 1000). After it was washed with fresh TBST, the membrane was incubated with HRP-conjugated, affinity-purified anti-rabbit IgG (0.1 *μ*g/mL). Immunoreactive bands were visualized using an enhanced chemiluminescence (ECL) kit (Pierce, USA) and an ECL Imaging System (Bio-Rad, USA). The signal intensities of the corresponding bands were quantified using Image-Pro Plus software.

### 2.11. Statistical Analysis

All values were tested for normal distribution and are expressed as the mean ± SD. The data were analyzed by one-way analysis of variance followed by a least significant difference test. *P* values < 0.05 were considered significant.

## 3. Results

### 3.1. Baicalin Decreased the mPAP in Rats with Hypoxic Pulmonary Hypertension

**Figure 1:**
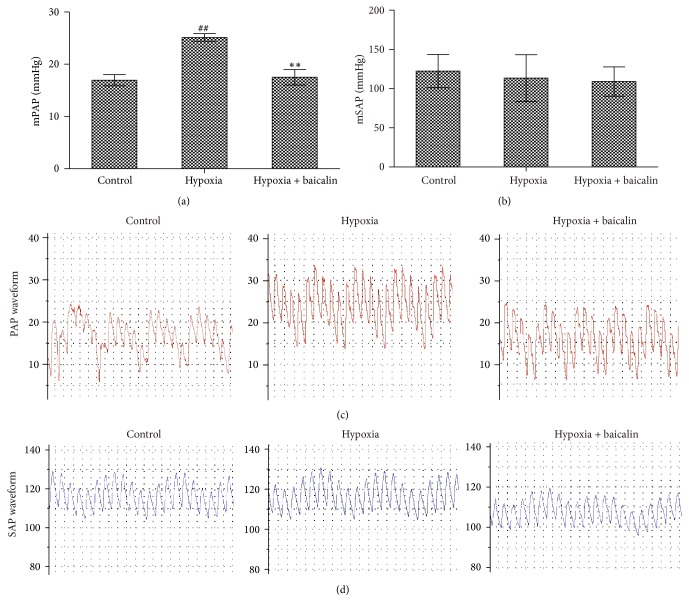
Effect of baicalin on pulmonary artery pressure in rats subjected to chronic hypoxia. (a, c) The mPAP was significantly increased in the hypoxia group but was decreased by baicalin. (*n* = 8/group) ## indicates *P* < 0.01 compared to the control group and *∗∗* indicates *P* < 0.01 compared to the hypoxia group. (b, d) No significant difference existed among the three groups.

The mSAP values of the control group and the hypoxia group were 122.35 ± 21.15 and 113.40 ± 29.86 mmHg, respectively, and the mean value after the baicalin treatment was 109.03 ± 18.73 mmHg. However, there were no significant differences among the three groups (Figures 1(b) and 1(d)). The mPAP was significantly higher in the hypoxia group than in the control group (25.12 ± 0.74 mmHg versus 16.94 ± 1.07 mmHg; *P* < 0.01), and the baicalin treatment remarkably reduced the mPAP to 17.50 ± 1.48 mmHg (*P* < 0.01) (Figures 1(a) and 1(c)).

### 3.2. Baicalin Attenuated the Pulmonary Arteriole and Right Ventricular Remodeling

**Figure 2:**
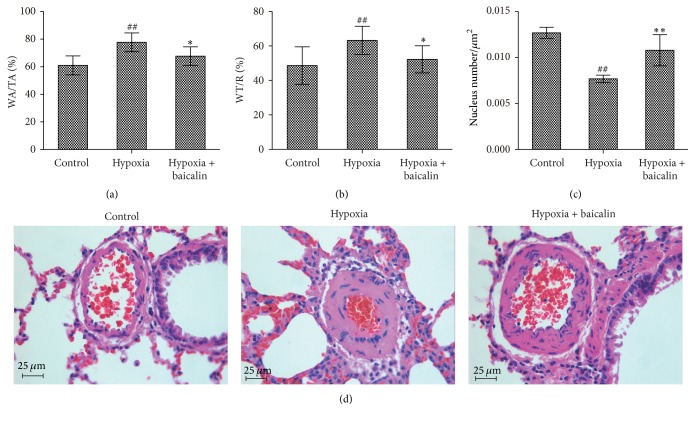
Effect of baicalin on pulmonary arteriole remodeling in rats subjected to chronic hypoxia. (a, b) The WA/TA and WT/R ratios were significantly increased in rats subjected to continuous hypoxia, but baicalin attenuated this trend. (*n* = 8/group) ## indicates *P* < 0.01 compared to the control group and *∗* indicates *P* < 0.05 compared to the hypoxia group. (c) The nuclei density significantly decreased under continuous hypoxia, and the trend was partially attenuated by baicalin. (*n* = 8/group) ## indicates *P* < 0.01 compared to the control group and *∗∗* indicates *P* < 0.01 compared to the hypoxia group. (d) Baicalin inhibited hypoxic pulmonary arteriole remodeling, as indicated by HE staining.

**Figure 3:**
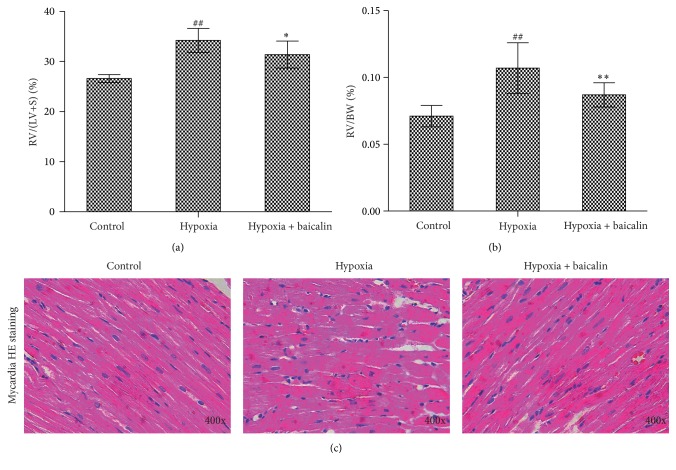
Effect of baicalin on myocardial remodeling in rats subjected to chronic hypoxia. (a, b) The RV/(LV + S) and RV/BW ratios were significantly increased in the hypoxia group, but baicalin attenuated this trend. (*n* = 8/group), ## indicates *P* < 0.01 compared to the control group, *∗* indicates *P* < 0.05 compared to the hypoxia group, and *∗∗* indicates *P* < 0.01 compared to the hypoxia group. (c) HE staining showed that chronic hypoxia increased right ventricular myocardial hypertrophy and cardiac muscle disorder but that baicalin partially attenuated these effects.

As shown in Figures 2(a) and 2(b), the WA/TA of the hypoxia group was greater than that of the control group (77.75 ± 6.79% versus 61.00 ± 6.86%; *P* < 0.01), and baicalin remarkably decreased the WA/TA to 67.72 ± 6.76% (*P* < 0.05). Similarly, the WT/*R* in the control group was 48.61 ± 10.92% but was 63.21 ± 8.19% in the hypoxia group (*P* < 0.01); baicalin attenuated this effect, as demonstrated by the reduced WT/*R* of 52.21 ± 7.86% (*P* < 0.05). In contrast, the vessel wall nuclear density in the control group was 0.0127 ± 0.0006/*μ*m^2^ but was 0.0077 ± 0.0004/*μ*m^2^ in the hypoxia group (*P* < 0.01); baicalin led to an increased nuclear density of 0.0108 ± 0.0017/*μ*m^2^ (*P* < 0.01) (Figure 2(c)). Hypoxic pulmonary arteriole remodeling was remarkably inhibited by baicalin, as visualized by H&E staining (Figure 2(d)). The RV/LV + S was 26.6 ± 0.8% in the control group and 34.2 ± 2.4% (*P* < 0.01) in the hypoxia group, but this parameter was considerably reduced to 31.4 ± 2.7% (versus the hypoxia group; *P* < 0.05) after the baicalin treatment (Figure 3(a)). As indicated in Figure 3(b), chronic hypoxia increased the RV/BW from 0.071 ± 0.008% in the control group to 0.107 ± 0.019% (*P* < 0.01), and baicalin decreased the RV/BW to 0.087 ± 0.009% (*P* < 0.01). Myocardial hypertrophy was present in the hypoxia group but was ameliorated by baicalin (Figure 3(c)).

### 3.3. Baicalin Downregulated p38 MAPK Activity

**Figure 4:**
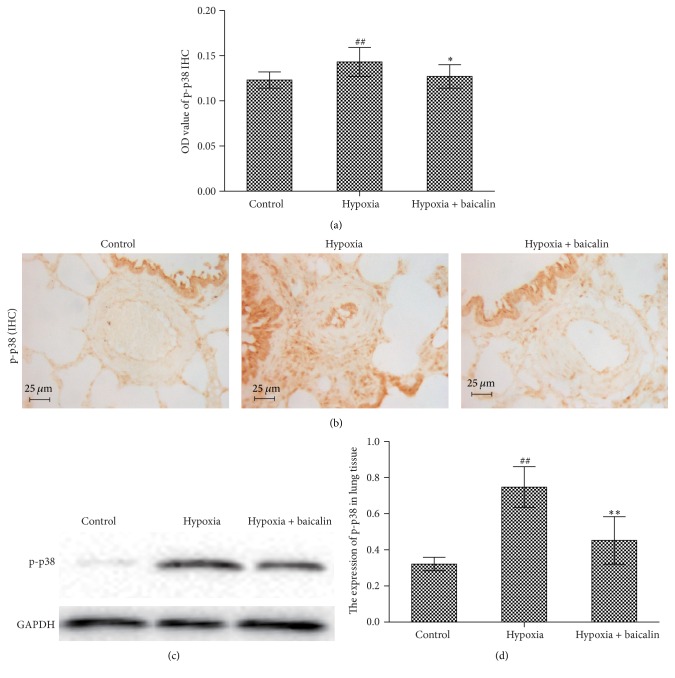
Expression of p-p38 in pulmonary arterioles and lung tissue. (a, b) p-p38 was expressed more abundantly in pulmonary arteriole cell nuclei in the hypoxia group than in the baicalin group and the control group, as visualized by immunohistochemistry. (*n* = 8/group) ## indicates *P* < 0.01 compared to the control group and *∗* indicates *P* < 0.05 compared to the hypoxia group. (c, d) Western blot analysis of p-p38 expression in the lung showed that p-p38 expression was higher in the hypoxia group than in the control group and that this increased expression was attenuated by baicalin treatment. Representative blots are shown. (*n* = 8/group) ## indicates *P* < 0.01 compared to the control group and *∗∗* indicates *P* < 0.01 compared to the hypoxia group.

Levels of the main active product of p38 MAPK, that is, p-p38 MAPK, were detected in the arteriole walls and lung tissue homogenates by immunohistochemistry and western blot analysis, respectively. In the hypoxia group, p-p38 MAPK was significantly increased in the arteriole walls, as indicated by the increase in OD from 0.122 ± 0.008 to 0.143 ± 0.016 (*P* < 0.01). The administration of baicalin significantly decreased this value to 0.127 ± 0.013 (*P* < 0.05) (Figures 4(a) and 4(b)). The analysis of p-p38 MAPK expression in the lung tissue homogenates showed the same trend. Compared with the control group, the hypoxia group showed increased p-p38 MAPK expression (0.3213 ± 0.0371 vs. 0.7474 ± 0.1130; *P* < 0.01), and baicalin significantly decreased this value to 0.4518 ± 0.1316 (*P* < 0.01) (Figures 4(c) and 4(d)).

### 3.4. Baicalin Downregulated MMP-9 Protein and mRNA Expression

**Figure 5:**
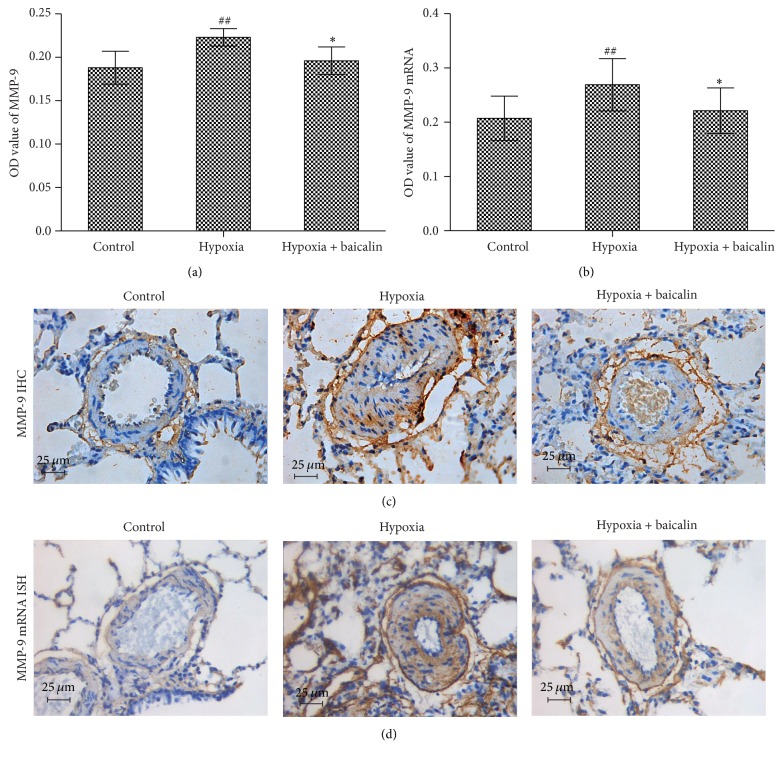
Effect of baicalin on MMP-9 protein and mRNA expression in pulmonary arterioles. (a, b) Immunohistochemistry and in situ hybridization techniques showed that both MMP-9 protein and mRNA levels were higher in the hypoxia group than in the control group and that baicalin attenuated this trend. (*n* = 8/group) ## indicates *P* < 0.01 compared to the control group and *∗* indicates *P* < 0.05 compared to the hypoxia group. (c) MMP-9 expression in pulmonary arterioles, as visualized by immunohistochemistry. (d) MMP-9 mRNA expression in pulmonary arterioles, as visualized by in situ hybridization.

Immunohistochemistry was used to detect MMP-9 protein expression in paraffin-embedded sections. Chronic hypoxia increased the OD value from 0.188 ± 0.019 in the control group to 0.223 ± 0.010 (*P* < 0.01), and the baicalin treatment decreased this value to 0.196 ± 0.016 (*P* < 0.05) (Figures 5(a) and 5(c)). MMP-9 mRNA expression was detected by ISH. The rat arteriole MMP-9 mRNA OD value was significantly elevated from 0.2073 ± 0.0413 to 0.2689 ± 0.0482 (*P* < 0.01) after exposure to hypoxia for 4 weeks, and this increase was significantly inhibited by the baicalin treatment, as indicated by the OD value reduction to 0.2206 ± 0.0423 (*P* < 0.05) (Figures 5(b) and 5(d)).

## 4. Discussion

Hypoxic pulmonary hypertension is a disease characterized by continuous increases in pulmonary vascular resistance and PAP. Pulmonary arteriole remodeling caused by a continuous state of hypoxia is the pathological basis of PAH, and the remodeling mechanism is complicated. ECM accumulation, fibroblast proliferation, and myofibroblast transformation, as well as smooth muscle cell proliferation, migration, and phenotypic differentiation, play key roles in this remodeling process [13].

Baicalin, a notable extract from* Scutellaria baicalensis*, has gained the attention of many scholars due to its antioxidative, anti-inflammatory, antitumor, antiapoptotic, antiproliferative, and antifibrotic activities. In previous studies, we have shown that baicalin attenuates hypoxia-induced pulmonary arteriole remodeling. However, this mechanism has yet to be fully elucidated.

The vascular ECM plays an important role in the formation of pulmonary arteries and contributes to the structural and functional integrity of the vasculature by providing the vessels with mechanical strength, elasticity, and compressibility. The ECM provides a microenvironment for cell-cell and cell-ECM communication to regulate vascular cell proliferation, migration, and differentiation and to sustain vascular homeostasis. As such, maintaining the composition of the ECM is important. The ECM mainly includes elastin, collagens, proteoglycans, structural glycoproteins, and adhesive glycoproteins, and the turnover of these components can cause an imbalance that eventually leads to vascular remodeling [14, 15]. MMP-9 is a member of the MMP family that plays a critical role in maintaining the balance of the ECM. A study using a hypoxia-induced pulmonary hypertension model in Sprague-Dawley rats showed that the enhanced expression of MMP-9 disturbed this balance and led to pulmonary remodeling [16].

As a response to hypoxic stimulation, pulmonary artery smooth muscle cells (PASMCs) proliferate and synthesize matrix material, which regulates the phenotype and proliferation of vascular smooth muscle cells (VSMCs), leading to increased vessel WT and abnormal muscularization of the normally nonmuscular distal pulmonary arteries. VSMC migration begins with lesion establishment and persists throughout the process of vascular remodeling. In addition, VSMC migration plays a significant role in neointima formation and the muscularization of distal pulmonary arteries. PASMCs in normal adult lung blood vessels are mostly quiescent [13, 17]. MMP-9, which is active in the process of arterial remodeling, plays a critical role in promoting VSMC proliferation and migration, as well as intimal thickening [18–20].

p38 MAPK belongs to the serine/threonine protein kinase family and plays a key role in the activation of proliferation [21, 22]. Chen et al. reported that the downregulation of p38 MAPK phosphorylation is associated with the effect of AVE0991 in attenuating angiotensin II-induced VSMC proliferation [23]. In balloon-injured rat aortas, VSMC proliferation and intimal thickening are reduced, which might be associated with the inhibition of the p38 MAPK signaling pathway [24]. p38 plays a role in attenuating cardiac muscle remodeling mainly by activating MMP-9 via an NAD(P)H-independent mechanism [6]. Cho et al. found that MMP-9 expression in arterial smooth muscle cells could be obviously inhibited by blocking p38 MAPK signaling [25]. In recent years, some studies have confirmed that blocking p38 MAPK signaling with specific blocker SB203580 can be used to inhibit MMP-9 expression in the vascular walls of small arteries and the aorta, for example, coronary arteries or the thoracic aorta, as well as improve vascular remodeling [26, 27]. Morin and his colleagues discovered that, in a monocrotaline-induced pulmonary hypertension model, pulmonary artery remodeling might be improved via downregulation of the activity of p38 MAPK and the expression of MMP-9 [28]. Li et al. found in their study that chronic hypoxia could stimulate endothelial cell migration and pulmonary artery smooth muscle cell proliferation to promote the formation of pulmonary hypertension via p38 MAPK signal pathway [29].

In our study, an experimental hypoxic chamber was used to simulate the hypoxic pulmonary state that exists in patients with chronic obstructive pulmonary disease or individuals living in regions with thin air. Compared to the control group, the hypoxia group showed significant increases in the mPAP, pulmonary arteriole WT, and cardiac hypertrophy indices, such as the RV/(LV + S) and RV/BW. Importantly, baicalin partially alleviated the negative effects induced by hypoxia. In this study, the MMP-9 mRNA and protein expression levels were significantly increased in the hypoxia group compared to the control group. Based on the role of MMP-9 in pulmonary arteriole remodeling, as confirmed by the literature, we concluded that the upregulation of MMP-9 mRNA and protein might also be involved in hypoxia-induced pulmonary hypertension. Importantly, the baicalin treatment attenuated the hypoxia-induced increases in MMP-9 and significantly improved the pulmonary arteriole remodeling. An analysis of the p-p38 MAPK levels in the arterioles and pulmonary tissue homogenates showed a trend similar to that of MMP-9, as the level was increased in the continuous hypoxia state but was suppressed by baicalin. These data also showed that pulmonary hypertension and the hypertrophy index were suppressed by baicalin, thus demonstrating that baicalin treatment can attenuate hypoxic cor pulmonale.

Baicalin clearly ameliorates pulmonary hypertension, pulmonary arterial remolding, and hypoxic cor pulmonale induced by hypoxia by inhibiting the p38 MAPK signaling pathway and MMP-9 expression. Considering the close relationship between p38 MAPK and MMP-9 that has been reported, the protective effect of baicalin may be achieved by inhibiting the p38 MAPK signaling pathway and suppressing MMP-9 expression.
